# The Severity of COVID-19 Is Associated with Greater Impairment of Cardiac Autonomic Modulation—Physical Training as a Countermeasure

**DOI:** 10.3390/jfmk11020149

**Published:** 2026-04-02

**Authors:** Noemi Marchini de Souza Couto, João Vitor Martins Bernal, Tábata de Paula Facioli, Daniel dos Santos, Hugo Celso Dutra de Souza

**Affiliations:** 1 Department of Health Sciences, Ribeirão Preto Medical School, University of São Paulo, Ribeirão Preto 14049-900, SP, Brazil; noemimarchini@hotmail.com (N.M.d.S.C.); fisio.joaomartins@gmail.com (J.V.M.B.); tabatafacioli@gmail.com (T.d.P.F.); 2 Department of Health Promotion, University of Franca, Franca 14404-600, SP, Brazil; daniel.santos@unifran.edu.br

**Keywords:** long COVID, autonomic cardiovascular control, aerobic physical training, cardiovascular adaptations

## Abstract

**Background:** COVID-19 has been associated with persistent impairments in autonomic modulation of heart rate variability (HRV). However, whether disease severity during the acute phase influences the magnitude of these impairments remains insufficiently explored. In turn, aerobic physical training (APT) has been proposed as a countermeasure to autonomic dysfunction of HRV in different conditions, although its effects in individuals with COVID-19 are not yet well established. To address these gaps, this study investigated the consequences of COVID-19 on autonomic modulation of HRV according to disease severity and evaluated the effects of APT on this parameter. **Methods:** One hundred and sixteen individuals (58 men and 58 women) aged between 30 and 55 years were included, allocated into three groups according to the severity of the disease in the acute phase: Mild group (*n* = 38, mean age: 48 ± 7 years); Moderate group (*n* = 52, mean age: 43 ± 5 years); and Severe group (*n* = 26, mean age: 45 ± 6 years). All groups had anthropometric and hemodynamic parameters evaluated before and after the 16-week APT period, as well as parameters of autonomic modulation of HRV analyzed using linear (time and frequency domain) and non-linear (symbolic analysis) methods obtained from R–R interval (RRi) recordings in the supine position for 30 min. **Results:** Initially, all groups presented similar anthropometric and hemodynamic values. In contrast, the Moderate and Severe groups presented lower values for standard deviation of normal RRi (SDNN; Moderate: 38 ± 14 ms; Severe: 33 ± 12 ms vs. Mild: 55 ± 28 ms; *p* < 0.001), root mean square difference between adjacent normal RRi (RMSSD; Moderate: 28 ± 13 ms; Severe: 22 ± 7 ms vs. Mild: 47 ± 38 ms; *p* < 0.001), total variance (Moderate: 203 ± 127 ms^2^; Severe: 303 ± 157 ms^2^ vs. Mild: 526 ± 347 ms^2^; *p* < 0.001), and high-frequency (HF) oscillations in absolute units (Moderate: 259 ± 270 ms^2^; Severe: 153 ± 74 ms^2^ vs. Mild: 438 ± 421 ms^2^; *p* < 0.001), both compared to the Mild group. In turn, the Severe group, when compared to the other groups, also presented lower HF oscillations (Severe: 29 ± 12 nu vs. Mild: 44 ± 17 nu and Moderate: 42 ± 17 nu; *p* < 0.001) and higher low-frequency (LF) oscillations (Severe: 71 ± 12 nu vs. Mild: 60 ± 17 nu and Moderate: 58 ± 17 nu; *p* < 0.001), but in normalized units. After the 16-week APT, all groups showed increases in HF oscillations (Mild: −206 ms^2^ and −19.12 nu; Moderate: −236 ms^2^ and −26.7 nu; Severe: −211 ms^2^ and −31.0 nu; *p* < 0.001) and reductions in LF oscillations (Mild: 198 ms^2^ and 19.01 nu; Moderate: 98 ms^2^ and 26.7 nu; Severe: 218 ms^2^ and 31.1 nu; *p* < 0.001), both in absolute and normalized units. In this case, there were no further differences in LF and HF oscillations between the groups. **Conclusions**: Individuals who had COVID-19 and developed moderate to severe cases showed greater impairments in the autonomic modulation of HRV, characterized by increased sympathetic autonomic modulation and reduced vagal modulation. In turn, APT as a countermeasure appears to increase vagal autonomic modulation and reduce sympathetic autonomic modulation of HRV, regardless of the previous severity of COVID-19.

## 1. Introduction

Long COVID, also referred to as post-COVID-19 syndrome, is a multisystem condition characterized by the persistence of signs and symptoms for more than 12 months after the acute phase of infection with SARS-CoV-2 [1]. Evidence indicates that approximately 10% to 30% of previously infected individuals develop prolonged symptoms [2,3,4]. Among the most prevalent manifestations are cardiovascular symptoms, including orthostatic intolerance, arrhythmias, and postural tachycardia, which are frequently associated with impairments in cardiovascular autonomic modulation [5,6,7,8].

More specifically, cardiovascular autonomic dysfunction in long COVID has been characterized by sympathetic hyperactivity, reduced heart rate variability (HRV), and impaired baroreflex sensitivity [9,10,11,12]. Taken together, these alterations define a profile of autonomic dysregulation associated with increased hemodynamic instability and elevated cardiovascular risk [13,14,15]. Although such manifestations have been observed even after mild acute disease, the heterogeneity of findings suggests that the severity of the initial infection may influence both the magnitude and the functional profile of these alterations during the post-infection period [10,16,17].

In this context, despite the growing body of evidence on autonomic dysfunction in long COVID, it remains unclear whether acute disease severity modulates the extent of these alterations and the response to therapeutic interventions. Aerobic physical training (APT), in turn, is widely recognized as an effective strategy in cardiovascular rehabilitation, promoting favorable adaptations in autonomic modulation across different populations [18,19,20,21,22,23,24]. However, its effects in individuals with long COVID, stratified according to the severity of the initial infection, have not yet been clearly established. Therefore, the present study aimed to investigate the consequences of COVID-19 on autonomic modulation of HRV by stratifying individuals according to disease severity, as well as to evaluate the effects of APT in this population.

## 2. Materials and Methods

### 2.1. Participants and Ethical Aspects

This was a non-randomized clinical trial approved by the Research Ethics Committee of the University of Franca (Protocol No. 5.446.463/2022). Participant screening and recruitment were conducted at the Physical Therapy Teaching Clinic of the University of Franca between August 2022 and March 2025. All volunteers were fully informed about the scientific, ethical, and legal aspects of the study and were advised that refusal to participate or withdrawal at any time would not result in any penalty or harm. After these clarifications, those who agreed to participate signed the written informed consent form.

Eligible participants were men and women aged 30 to 55 years, previously diagnosed with COVID-19, with diagnosis confirmed by the presentation of positive laboratory test results for SARS-CoV-2 (reverse transcription–polymerase chain reaction [RT-PCR] or rapid antigen tests [Ag-RDT]) performed during the acute phase of the disease, and evaluated between 3 and 24 months after the quarantine period and/or hospital discharge. The presence of long COVID was defined as the persistence of one or more self-reported symptoms for at least three months after acute SARS-CoV-2 infection, including fatigue, lower limb pain, insomnia, dyspnea, hair loss, malaise, chest tightness, memory impairment, difficulty concentrating, cough, and episodes of tachycardia.

In turn, individuals who were smokers, regularly physically active, or previously submitted to home-based or outpatient physiotherapeutic treatment, as well as those with cognitive disorders, musculoskeletal impairments, hemodynamic instability, cardiovascular diseases, diabetes, or any other clinical condition that could compromise the performance of the tests or aerobic physical training, were excluded. Participants who did not attend the initial evaluation or did not complete the proposed protocol were also excluded.

After applying the eligibility criteria, the study sample comprised 116 participants (58 men and 58 women), who were allocated according to the severity of the clinical condition presented during the acute phase of COVID-19, considering the need for hospitalization, ventilatory support, and clinical follow-up during infection. Accordingly, participants were distributed into three groups: the Severe group, composed of individuals who required hospitalization and ventilatory support (*n* = 26; 18 men and 8 women; mean age: 45 ± 6 years); the Moderate group, consisting of individuals who presented clinically relevant pneumonia symptoms requiring medical follow-up but without hospitalization (*n* = 52; 24 men and 28 women; mean age: 43 ± 5 years); and the Mild group, formed by individuals with mild symptoms or asymptomatic presentation, without the need for hospital care (*n* = 38; 16 men and 22 women; mean age: 48 ± 7 years).

### 2.2. Experimental Protocols

Participants were instructed in advance to refrain from intense physical activity, avoid the consumption of alcoholic and caffeinated beverages for 36 h prior to testing, and to sleep between 7 and 8 h on the night preceding the evaluation. Before and after a 16-week period of aerobic physical training (APT), all participants underwent the following experimental protocols: anthropometric assessment, evaluation of basal cardiovascular hemodynamic parameters, beat-to-beat R–R interval (RRi) recording, and assessment of cardiorespiratory fitness through cardiopulmonary exercise testing. All evaluations were conducted in the morning (7:00 a.m. to 10:00 a.m.) during a single visit.

#### 2.2.1. Assessment of Anthropometric and Hemodynamic Parameters

Body mass and height were obtained using an analog scale with an attached stadiometer (Welmy; Santa Bárbara d’Oeste, São Paulo, Brazil). Body mass index (BMI) was calculated using the formula weight/height^2^, with weight expressed in kilograms and height in meters. Resting blood pressure was measured using the auscultatory method, using a properly calibrated aneroid sphygmomanometer (G-Tech, Zhuhai, China) and a stethoscope (Littmann Classic III, 3M, St. Paul, MN, USA). Measurements were performed with participants at rest, and two consecutive readings were obtained at 1 min intervals; the mean value was used for analysis.

#### 2.2.2. HRV Autonomic Modulation

The HRV was obtained using the RRi from the electrocardiographic recording (Dual Bio Amp/Stimulator, ADInstruments, Bella Vista, Australia), using a modified CM5 shunt at a sampling frequency of 500 Hz. A 30 min resting recording was performed with participants in the supine position; the first 10 min were considered an adaptation period, and the subsequent 20 min were used for HRV analysis. Ambient temperature (22 °C), lighting, and noise were strictly controlled. Autonomic modulation of HRV based on RRi was analyzed using linear methods (time and frequency domains) and a non-linear method. Time-domain analysis included the calculation of the root mean square of successive differences between normal RRi (RMSSD) and the standard deviation of normal RRi (SDNN), reflecting predominantly vagal and overall autonomic modulation, respectively. Frequency-domain analysis was performed using spectral analysis based on the fast Fourier transform (FFT), decomposing the signal into low-frequency (LF; 0.04–0.15 Hz) and high-frequency (HF; 0.15–0.40 Hz) components, expressed in absolute units (ms^2^) and normalized units (nu). HF components in both absolute (ms^2^) and normalized (nu) units were interpreted as indices of vagal autonomic modulation of HRV, whereas the LF component, particularly in normalized units (nu), was considered an index of sympathetic autonomic modulation. The LF/HF ratio was also calculated as an indicator of sympathovagal balance. Additionally, symbolic analysis, a non-linear method of HRV assessment, was applied. In this approach, patterns composed of three consecutive RR intervals were classified into four categories: 0V% (percentage of sequences without variation), 1V% (percentage of sequences with one variation), 2LV% (percentage of sequences with two like variations), and 2UV% (percentage of sequences with two unlike variations) [25,26,27,28].

#### 2.2.3. Assessment of Cardiorespiratory Fitness

Cardiorespiratory fitness was assessed at the Exercise Physiology and Cardiovascular Physical Therapy Laboratory (LAPHY-CARPHY) of the Ribeirão Preto Medical School, University of Sao Paulo, Brazil, using an incremental cardiopulmonary exercise test (CPET) performed on a motorized cycle ergometer (E5, COSMED, Rome, Italy), following the Balke protocol [29]. The test was terminated upon volitional exhaustion or according to clinical and hemodynamic criteria, including chest pain or discomfort, disproportionate dyspnea relative to exercise intensity, systolic blood pressure (SBP) > 220 mmHg or diastolic blood pressure (DBP) > 120 mmHg, occurrence of persistent arrhythmias during exercise, absence of an increase or a decrease in SBP during exertion, or attainment of submaximal heart rate (HR), defined as 90% of HR reserve calculated by subtracting resting HR from the estimated maximal HR determined by the equation 208 − (0.7 × age) [30]. Ventilatory and metabolic parameters were obtained using a computerized metabolic system (Quark CPET, COSMED, Rome, Italy). During the test, perceived exertion was also assessed using the modified Borg scale [31]. Maximal effort was confirmed based on respiratory exchange ratio (RER) ≥ 1.10 [32].

### 2.3. Training Protocol

After the baseline assessment, participants underwent a supervised and monitored continuous APT protocol performed on a cycle ergometer at individualized submaximal intensity, corresponding to the HR within ±5% of the anaerobic threshold determined during CPET. The training program lasted 16 weeks, with a minimum attendance requirement of 85%, and consisted of three sessions per week, each lasting 60 min. Each session was structured into four phases: warm-up (5 min) at an intensity below the target HR; continuous APT (40 min) at the target HR; recovery period (5 min); and general stretching exercises (10 min). During the first two weeks, the APT phase lasted between 20 and 30 min to allow familiarization and adaptation to the protocol. In this initial phase, training intensity was set at an HR corresponding to −10% of the HR at the anaerobic threshold, with weekly progression of both intensity and duration until the target training HR was achieved.

Training sessions were supervised by trained physiotherapists, with continuous HR monitoring during all sessions using chest-strap HR monitors (Polar H10). All APT sessions were conducted at the Physical Therapy Teaching Clinic of the University of Franca, Sao Paulo, Brazil.

### 2.4. Statistical Analysis

Sample size was estimated using power analysis performed with GraphPad software (version 10.6.1, GraphPad Software, San Diego, CA, USA). The calculation considered HRV parameters, specifically LF and HF oscillations expressed in normalized units, as the primary outcomes, assuming a significance level of 5% and a statistical power of 80%.

Data were analyzed using the Jamovi statistical software (version 2.3; Jamovi, Sydney, Australia). Data normality was assessed using the Shapiro-Wilk test. Continuous variables were described as mean ± standard deviation (SD) and, when appropriate, accompanied by 95% confidence intervals. Comparisons were performed using parametric tests, and non-parametric tests were applied when required. Intragroup comparisons were conducted using the paired *t*-test or the Wilcoxon test, as appropriate. Intergroup comparisons, with groups categorized according to infection severity (mild, moderate, and severe), were performed using one-way analysis of variance (ANOVA), followed by Tukey’s post hoc test. For non-parametric variables, the Kruskal–Wallis test was applied, followed by Dunn’s post hoc test. The level of statistical significance was set at *p* < 0.05 for all analyses.

## 3. Results

**Figure 1:**
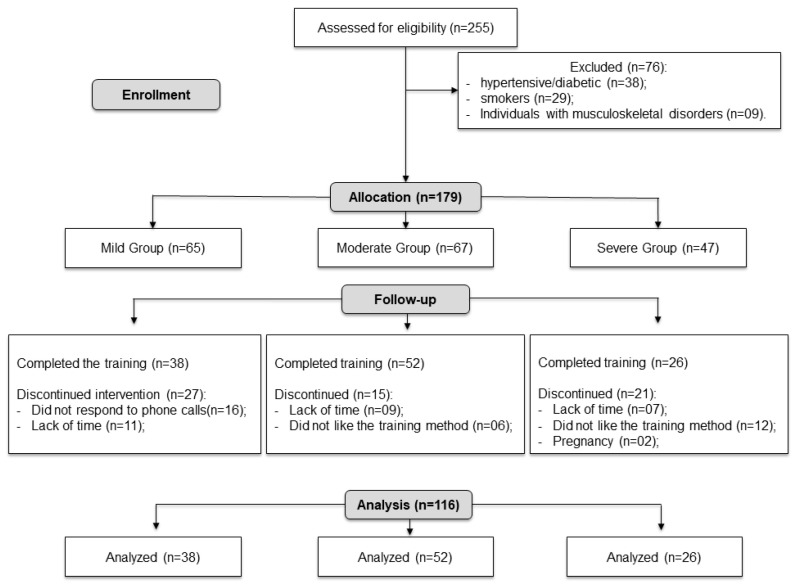
Participant flow diagram.

A total of 255 individuals were assessed for eligibility. After applying the predefined eligibility criteria, 76 participants were excluded (38 with hypertension or diabetes, 29 smokers, and 9 with musculoskeletal disorders), and 179 were allocated to the study groups (Mild Group, *n* = 65; Moderate Group, *n* = 67; Severe Group, *n* = 47). During the follow-up period, some participants discontinued the intervention due to lack of availability, lack of interest in the training protocol, or other personal reasons. A final sample of 116 participants completed the study and were included in the analysis. The detailed flow of participants throughout the study is presented in Figure 1.

Table 1 presents the data as mean ± standard deviation (SD), whereas Table 2 shows the intragroup comparisons of anthropometric characteristics and cardiovascular and metabolic parameters before and after APT. All groups exhibited reductions in body weight and waist-to-hip ratio, while a reduction in BMI was observed only in the Moderate and Severe groups. In addition, APT promoted decreases in HR, SBP, DBP, and mean blood pressure (MBP), as well as increases in peak oxygen consumption (VO_2peak_) and metabolic equivalent (MET) values in all groups.

Table 3 presents the data as mean ± standard deviation (SD) before APT. In the time-domain HRV analysis prior to APT (Table 3), the Moderate and Severe groups exhibited lower SDNN and RMSSD values compared with the Mild group. In the frequency domain, the Moderate and Severe groups also showed lower values of total variance and HFms^2^, whereas only the Moderate group presented lower LFms^2^ values compared with the Mild group. In addition, the Severe group exhibited higher LFnu values and a higher LF/HF ratio, as well as lower HFnu values, compared with the Mild and Moderate groups.

Table 4 presents the intragroup comparisons of cardiovascular autonomic parameters before and after APT. In the time-domain HRV analysis, all groups exhibited a significant increase in RRi values after the intervention. Similarly, significant increases in SDNN were observed in the Moderate and Severe groups, whereas an increase in RMSSD was observed only in the Moderate group. In the frequency domain, APT promoted a significant increase in total variance in all groups. In addition, LFms^2^ oscillations were reduced in the Mild, Moderate, and Severe groups, whereas HFms^2^ oscillations increased in all groups. When expressed in normalized units, all groups showed reductions in LFnu accompanied by concomitant increases in HFnu, along with a significant decrease in the LF/HF ratio. No significant changes were observed in symbolic analysis parameters in any of the groups.

Table 5 presents the data as mean ± standard deviation (SD) for cardiovascular autonomic parameters after 16 weeks of APT. In the time-domain HRV analysis, the Severe group exhibited lower RRi values compared with the Mild group. No significant differences were observed among groups for SDNN and RMSSD. In the frequency domain, the Moderate and Severe groups showed lower total variance values compared with the Mild group. No significant differences were observed among groups for LFms^2^, HFms^2^, LFnu, HFnu, the LF/HF ratio, 0V%, or 2UV%.

## 4. Discussion

The present study aimed to investigate the consequences of COVID-19 on the autonomic modulation of HRV, stratifying individuals according to disease severity, as well as to evaluate the effects of APT in this population. Our main findings demonstrate that individuals in the moderate and severe groups exhibited greater impairments in HRV autonomic modulation compared with those in the mild group. In turn, when subjected to APT, all groups showed beneficial adaptations in HRV autonomic modulation, characterized by increased vagal autonomic modulation and reduced sympathetic autonomic modulation, regardless of COVID-19 severity.

Previous studies have demonstrated the persistence of cardiovascular autonomic dysfunction in individuals affected by COVID-19, irrespective of the severity of the initial infection [10,16,17]. Our findings suggest that the magnitude of this impairment is not homogeneous across different clinical phenotypes. In this context, individuals who experienced moderate or severe clinical presentations during the acute phase of COVID-19 exhibited lower HRV variance and reduced vagal autonomic modulation compared with those who experienced mild acute disease, indicating a greater impairment in autonomic regulation that appears to correlate with the severity of the acute phase of COVID-19. These differences may be explained, at least in part, by a greater residual inflammatory response in individuals who developed more severe forms of COVID-19. In fact, previous evidence indicates that chronic low-grade inflammation, which is more prevalent in moderate and severe clinical conditions, may promote persistent sympathetic hyperactivity and reduced vagal modulation, thereby contributing to impairments in cardiovascular autonomic regulation [33,34,35,36]. Therefore, our findings add to the existing literature by demonstrating that the severity of acute infection is associated with the extent of autonomic modulation impairment observed in individuals affected by COVID-19.

In this context, APT represents an effective therapeutic strategy to attenuate these impairments, promoting favorable adaptations in HRV autonomic modulation across all groups. These benefits may be associated with positive changes in anthropometric, hemodynamic, and metabolic parameters after 16 weeks of APT, such as reductions in resting HR and blood pressure, as well as improvements in cardiorespiratory fitness (VO_2peak_), irrespective of disease severity. These adaptations reflect enhanced cardiovascular efficiency, which is closely linked to beneficial autonomic adjustments, characterized by increased vagal modulation and reduced sympathetic modulation of HRV. Furthermore, the reductions in BMI and waist-to-hip ratio observed in the moderate and severe groups suggest an attenuation of pro-inflammatory and sympatho-excitatory mechanisms [37,38,39]. Previous evidence indicates that APT, particularly in individuals with cardiovascular and metabolic diseases, promotes greater efficiency in cardiovascular autonomic regulation, characterized by increased HRV, reduced sympathetic dependence, enhanced baroreflex sensitivity, and anti-inflammatory and antioxidant effects, in addition to improved endothelial function. Collectively, these adaptations may act synergistically to partially restore cardiovascular autonomic balance [23,40] and are also reflected in a more efficient cardiorespiratory response to exercise, as assessed by CPET, reinforcing its value in the comprehensive evaluation of cardiovascular function and risk [41].

## 5. Conclusions

Individuals who had COVID-19 and developed moderate to severe cases showed greater impairments in the autonomic modulation of HRV, characterized by increased sympathetic autonomic modulation and reduced vagal modulation. In turn, APT as a countermeasure appears to increase vagal autonomic modulation and reduce sympathetic autonomic modulation of HRV, regardless of the previous severity of COVID-19.

## 6. Clinical Implications, Limitations, and Future Directions

This study has relevant clinical implications, as reduced HRV is closely associated with increased cardiovascular risk [13,14,15]. In this context, our findings indicate that APT constitutes an effective therapeutic strategy for individuals presenting chronic complications related to COVID-19, regardless of the severity of the previous infection. 

Despite its relevance, this study presents some limitations that should be considered when interpreting the results. The analysis was performed without stratification by sex, which may have influenced HRV outcomes, although the proportion of men and women was similar across groups. In addition, the absence of catecholamine measurements, as well as assessments of endothelial function and inflammatory and oxidative stress markers, may have limited a more comprehensive understanding of the pathophysiological mechanisms underlying the observed adaptations.

## Figures and Tables

**Table 1:** Characteristics and hemodynamic and metabolic parameters obtained before and after the 16 weeks of aerobic physical training in the Mild (*n* = 38), Moderate (*n* = 52) and Severe (*n* = 26) groups.

	Mild		Moderate		Severe	
	Before	After	Before	After	Before	After
**Characteristics**						
Age, years	48 (7)	-	43 (5)	-	45 (6)	-
Height, m	1.67 (0.11)	-	1.70 (0.9)	-	1.72 (0.10)	-
Weight, kg	82.7 (13.7)	81.7 (14.8)	84.5 (15.6)	84.5 (17.2)	92.7 (9.4)	91.0 (9.1)
BMI, kg/m^2^	29.5 (2.9)	29.3 (3.2)	29.0 (3.9)	28.5 (3.8)	31.2 (3.3)	30.5 (3.2)
**Baseline Cardiovascular Values**						
HR, bpm	78 (8)	71 (5)	78 (8)	73 (7)	81 (7)	73 (5)
SBP, mmHg	122 (9)	114 (10)	122 (13)	113 (12)	130 (7)	119 (13)
DBP, mmHg	79 (6)	75 (6)	80 (7)	73 (6)	90 (12)	76 (4)
MBP, mmHg	96 (7)	90 (7)	94 (9)	87 (8)	103 (10)	91 (6)
**Metabolic Values**						
VO_2peak_, mL/kg/min	14.0 (3.0)	21.0 (5.0)	13.8 (4.0)	19.5 (5.0)	13.5 (3.0)	19.1 (4.0)
MET, mL/kg/min	4.0 (0.9)	6.0 (1.5)	3.9 (1.2)	5.6 (1.4)	3.9 (0.8)	5.4 (1.2)

Values are expressed as mean (standard deviation); BMI, body mass index; HR, heart rate; SBP, systolic blood pressure; DBP, diastolic blood pressure; MBP, mean blood pressure; VO_2peak_, peak oxygen consumption during exercise; MET, metabolic equivalent; bpm, beats per minute; mmHg, millimeters of mercury; m, meters; kg, kilograms; mL/kg/min, milliliters per kilogram per minute.

**Table 2:** Intragroup comparison of characteristics and hemodynamic and metabolic parameters obtained before and after the 16 weeks of aerobic physical training in the Mild (*n* = 38), Moderate (*n* = 52) and Severe (*n* = 26) groups.

	Mild			Moderate			Severe		
	Estimation of Difference (CI 95%)	Statistic	*p*	Estimation of Difference (CI 95%)	Statistic	*p*	Estimation of Difference (CI 95%)	Statistic	*p*
**Characteristics**									
Weight, kg	0.90 (0.14; 1.66)	t(39) = 2.40	**0.021**	0.79 (0.02; 1.52)	W = 916	0.039 *	1.45 (1.00; 1.90)	W = 324.0	<0.001 *
BMI, kg/m^2^	0.23 (−0.07; 0.52)	t(39) = 1.54	0.131	0.50 (0.27; 0.74)	W = 1037	<0.001 *	0.50 (0.39; 0.66)	W = 351.0	<0.001 *
WHR	0.001 (−0.001; 0.01)	W = 260	0.031 *	0.020 (0.015; 0.020)	W = 673	<0.001 *	0.02 (0.009; 0.02)	W = 78.0	<0.001 *
**Baseline Cardiovascular Values**									
HR, bpm	6.42 (5.00; 8.00)	W = 820	<0.001 *	5.58 (4.50; 6.58)	W = 1325	<0.001 *	7.99 (6.00; 9.00)	W = 351.0	<0.001 *
SBP, mmHg	8.07 (6.35; 9.80)	t(39) = 9.47	**<0.001**	8.00 (7.0; 10.0)	W = 1275	<0.001 *	7.13 (5.99; 9.00)	W = 351.0	<0.001 *
DBP, mmHg	5.00 (4.00; 7.00)	W = 542	<0.001 *	6.00 (5.0; 7.0)	W = 1378	<0.001 *	11.00 (9.00; 12.00)	W = 351.0	<0.001 *
MBP, mmHg	5.82 (4.60; 7.03)	t(39) = 9.68	**<0.001**	6.67 (6.0; 8.0)	W = 1378	<0.001 *	9.33 (8.33; 10.66)	W = 351.0	<0.001 *
**Metabolic Values**									
VO_2peak_,mL/kg/min	−6.75 (−7.98; −5.91)	W = 0	<0.001 *	−5.34 (−5.93; −4.87)	W = 0	<0.001 *	−5.25 (−6.19; −4.85)	W = 0	<0.001 *
MET, mL/kg/min	−1.92 (−2.28; −1.69)	W = 0	<0.001 *	−1.53 (−1.69; −1.39)	W = 0	<0.001 *	−1.50 (−1.76; −1.38)	W = 0	<0.001 *

The data are presented as means and confidence intervals (CI 95%) with their respective minimum and maximum values. Estimation of difference was calculated as before − after; negative values indicate an increase after the intervention. d.f, degrees of freedom; BMI, body mass index; WHR, waist-to-hip ratio; HR, heart rate; SBP, systolic blood pressure; DBP, diastolic blood pressure; MBP, mean blood pressure; VO_2peak_, peak oxygen consumption during exercise; MET, metabolic equivalent; bpm, beats per minute; mmHg, millimeters of mercury. Parametric variables were analyzed using paired *t*-test and are presented as t(d.f). Nonparametric variables were analyzed using Wilcoxon signed-rank test and are presented as W; *, Wilcoxon test; Highlighted in bold: *p* < 0.05.

**Table 3:** Cardiovascular autonomic parameters obtained before 16 weeks of aerobic physical training in the Mild (*n* = 38), Moderate (*n* = 52) and Severe (*n* = 26) groups.

	Mild	Moderate	Severe	Statistic	*p*
**HRV—Time Domain**					
RRi, ms	874 (134)	841 (105)	819 (60)	F(2, 71) = 1.94	0.378 *
SDNN, ms	55 (28)	38 (14) *^a^*	33 (12) *^a^*	H(2) = 20.55	<0.001 *
RMSSD, ms	47 (38)	28 (13) *^a^*	22 (7) *^a^*	H(2) = 18.08	<0.001 *
**HRV—Frequency Domain**					
Variance, ms^2^	526 (347)	203 (127) *^a^*	303 (157) *^a,b^*	H(2) = 34.93	<0.001 *
LF, ms^2^	470 (202)	397 (438) *^a^*	440 (216) *^b^*	H(2) = 16.00	<0.001 *
HF, ms^2^	438 (421)	259 (270) *^a^*	153 (74) *^a^*	H(2) = 27.09	<0.001 *
LF, nu	60 (17)	58 (17)	71 (12) *^a,b^*	F(2, 69) = 10.67	**<0.001**
HF, nu	44 (17)	42 (17)	29 (12) *^a,b^*	F(2, 69) = 10.67	**<0.001**
LF/HF ratio	1.7 (1.3)	2.3 (2.9)	3.1 (1.6) *^a,b^*	H(2) = 14.69	<0.001 *
**HRV—Symbolic Analysis**					
0V%	36.7 (19.7)	34.7 (17.4)	43.1 (10.5)	H(2) = 5.64	0.060 *
2UV%	21.8 (12.4)	20.9 (11.2)	17.01 (7.2)	H(2) = 1.78	0.410 *

Data are presented as mean (standard deviation). d.f, degrees of freedom; HRV, heart rate variability; RRi, R–R interval; SDNN, standard deviation of normal RRi; RMSSD, root mean square difference between adjacent normal RRi; LF, low-frequency oscillations; HF, high-frequency oscillations; nu, normalized units; LF/HF, low- to high-frequency ratio; 0V%, symbolic patterns with no variation (sympathetic modulation); 2UV%, symbolic patterns with two unlike variations (vagal modulation). Parametric variables were analyzed using one-way ANOVA and are presented as F(d.f_1_, d.f_2_). Nonparametric variables were analyzed using the Kruskal–Wallis test and are presented as H(d.f). *^a^*, *p* < 0.05 vs. Mild Group; *^b^*, *p* < 0.05 vs. Moderate Group; *, Kruskal–Wallis test. Highlighted in bold: *p* < 0.05.

**Table 4:** Intragroup comparison of cardiovascular autonomic parameters obtained before and after the 16 weeks of aerobic physical training in the Mild (*n* = 38), Moderate (*n* = 52) and Severe (*n* = 26) groups.

	Mild			Moderate			Severe		
	Estimation of Difference (CI 95%)	Statistic	*p*	Estimation of Difference (CI 95%)	Statistic	*p*	Estimation of Difference (CI 95%)	Statistic	*p*
**HRV—Time Domain**									
RRi, ms	−100.4 (−137.4; −74.0)	W = 15	<0.001 *	−87.1 (−124.9; −57.5)	W = 31	<0.001 *	−97.8 (−128.6; −79.6)	W = 4.5	<0.001 *
SDNN, ms	−10.13 (−23.78; 3.52)	t(37) = −1.40	0.141	−10.99 (−15.19; −7.42)	W = 205	<0.001 *	−12.24 (−16.61; −8.35)	W = 0	<0.001 *
RMSSD, ms	−8.45 (−24.23; 7.33)	t(37) = −0.98	0.285	−7.87 (−14.27; −1.48)	t(51) = −2.36	**0.017**	−4.62 (−10.36; 0.82)	W = 115	0.085 *
**HRV—Frequency Domain**									
Variance, ms^2^	−429 (−604; −280)	W = 48	<0.001 *	−147 (−192; −120)	W = 0	<0.001 *	−163 (−223; −105)	W = 7	<0.001 *
LF, ms^2^	198 (156; 244)	W = 741	<0.001 *	98 (−163.6; 61.8)	W = 1156	<0.001 *	218 (127.6; 316.7)	W = 340	<0.001 *
HF, ms^2^	−206 (−419; −111)	W = 186	0.004 *	−236 (−268; −204)	t(51) = −15.88	**<0.001**	−211 (−274.7; −156.7)	W = 0	<0.001 *
LF, nu	19.01 (10.60; 26.66)	W = 619	<0.001 *	26.7 (22.2; 31.3)	t(51) = 11.72	**<0.001**	31.1 (23.4; 39.0)	t(25) = 8.58	**<0.001**
HF, nu	−19.12 (−26.66; −11.01)	W = 122	<0.001 *	−26.7 (−31.3; −22.1)	t(51) = −11.72	**<0.001**	−31.0 (−39.8; −21.1)	W = 0	<0.001 *
LF/HF ratio	0.76 (−0.45; 1.09)	W = 603	<0.001 *	1.12 (0.83; 1.51)	W = 1347	<0.001 *	2.06 (1.44; 2.84)	W = 351	<0.001 *
**HRV—Symbolic Analysis**									
0V%	−4.75 (−12.30; 2.70)	W = 314	0.401 *	−0.55 (−7.10; 5.00)	W = 661	0.809 *	−3.00 (−4.90; 0.95)	W = 117	0.098 *
2UV%	2.20 (−1.45; 6.50)	W =401	0.472 *	−0.754 (−3.80; 1.55)	W = 632	0.570 *	−1.77 (−5.53; 1.98)	t(25) = −0.881	0.341

Data are presented as means of the confidence intervals (CI 95%) with their respective minimum and maximum values. Estimation of difference was calculated as before − after; negative values indicate an increase and positive values indicate a reduction after the intervention. d.f, degrees of freedom; HRV, heart rate variability; RRi, R–R interval; SDNN, standard deviation of normal RRi; RMSSD, root mean square difference between adjacent normal RRi; LF, low-frequency oscillations; HF, high-frequency oscillations; nu, normalized units; LF/HF, low- to high-frequency ratio; 0V%, symbolic patterns with no variation (sympathetic modulation); 2UV%, symbolic patterns with two unlike variations (vagal modulation). Parametric variables were analyzed using paired *t*-test and are presented as t(d.f). Nonparametric variables were analyzed using Wilcoxon signed-rank test and are presented as W; * Wilcoxon test; Highlighted in bold: *p* < 0.05.

**Table 5:** Cardiovascular autonomic parameters obtained after 16 weeks of aerobic physical training in the Mild (*n* = 38), Moderate (*n* = 52) and Severe *(n* = 26) groups.

	Mild	Moderate	Severe	Statistic	*p* Value
**HRV—Time Domain**					
RRi, ms	983 (115)	938 (134)	930 (101) *^a^*	H(2) = 6.520	0.038 *
SDNN, ms	65.7 (42)	49.3 (21)	46 (16)	H(2) = 2.607	0.272 *
RMSSD, ms	55 (59)	36 (23)	27 (13)	H(2) = 4.522	0.104 *
**HRV—Frequency Domain**					
Variance, ms^2^	1058 (805)	452 (388) *^a^*	512 (353) *^a^*	H(2) = 25.788	<0.001 *
LF, ms^2^	259 (164)	256 (309)	204 (103)	H(2) = 4.298	0.117 *
HF, ms^2^	703 (656)	458 (198)	387 (191)	H(2) = 1.856	0.395 *
LF, nu	39 (25)	31 (15)	39 (19)	H(2) = 1.754	0.416 *
HF, nu	61 (25)	69 (15)	61 (19)	H(2) = 1.754	0.416 *
LF/HF ratio	1.0 (1.1)	0.57 (0.58)	0.83 (0.7)	H(2) = 1.754	0.416 *
**HRV—Symbolic Analysis**					
0V%	42.8 (19.6)	36.8 (16)	42.8 (13.9)	H(2) = 2.338	0.311 *
2UV%	18.5 (9.3)	21.3 (11.9)	18.7 (9.9)	H(2) = 0.498	0.779 *

Data are presented as mean (standard deviation). d.f, degrees of freedom; HRV, heart rate variability; RRi, R–R interval; SDNN, standard deviation of normal RRi; RMSSD, root mean square difference between adjacent normal RRi; LF, low-frequency oscillations; HF, high-frequency oscillations; nu, normalized units; LF/HF, low- to high-frequency ratio; 0V%, symbolic patterns with no variation (sympathetic modulation); 2UV%, symbolic patterns with two unlike variations (vagal modulation). Parametric variables were analyzed using one-way ANOVA and are presented as F(d.f_1_, d.f_2_). Nonparametric variables were analyzed using the Kruskal–Wallis test and are presented as H(d.f). *^a^*, *p* < 0.05 vs. Mild Group; *, Kruskal–Wallis test. Highlighted in bold: *p* < 0.05.

## Data Availability

The data supporting the conclusions of this study are available upon reasonable request to the corresponding author.

## Abbreviations

The following abbreviations are used in this manuscript:

COVID-19	Coronavirus Disease-2019
HRV	Heart Rate Variability
APT	Aerobic Physical Training
RRi	R-R intervals beat by beat
SDNN	Standard Deviation of Normal RRi
RMSSD	Square Root of the Mean of the Square of the Differences between Adjacent Normal RRi
HF	High-Frequency Oscillations
LF	Low-Frequency Oscillations
BMI	Body Mass Index
FFT	Fast Fourier Transform
SBP	Systolic Blood Pressure
DBP	Diastolic Blood Pressure
MBP	Mean Blood Pressure
HR	Heart Rate
SD	Standard Deviation
VO_2peak_	Peak Oxygen Consumption
MET	Metabolic Equivalent
HFms^2^	High-Frequency Oscillations in Absolute Values
LFms^2^	Low-Frequency Oscillations in Absolute Values
HFnu	High-Frequency Oscillations in Normalized Values
LFnu	Low-Frequency Oscillations in Normalized Values
RER	Respiratory Exchange Ratio
